# Plasma Concentration of Taurine Changes following Acetaminophen Overdose in Male Patients during Hospitalization 

**DOI:** 10.22037/ijpr.2020.113698.14435

**Published:** 2021

**Authors:** Mohammadreza Sattari, Ali Ostadi, Shokoufeh Hassani, Zeynab Mazloumi, Hamid Noshad, Kayvan Mirnia, Armin Salek Maghsoudi

**Affiliations:** a *Liver and Gastrointestinal Diseases Research Center, Tabriz University of Medical Sciences, Tabriz, Iran. *; b *Department of Pharmacology and Toxicology, Faculty of Pharmacy, Tabriz University of Medical Sciences, Tabriz, Iran. *; c *Department of Internal Medicine, Sina Hospital, Tabriz University of Medical Sciences, Tabriz, Iran. *; d *Toxicology and Diseases Group (TDG), Pharmaceutical Sciences Research Center (PSRC), The Institute of Pharmaceutical Sciences (TIPS), Tehran University of Medical Sciences, Tehran, Iran. *; e *Department of Toxicology and Pharmacology, Faculty of Pharmacy, Tehran University of Medical Sciences, Tehran, Iran. *; f *Department of Pharmacology and Toxicology, Faculty of Pharmacy, Mazandaran University of Medical Sciences, Sari, Iran. *; g *Department of Nephrology, Sina Hospital, Tabriz University of Medical Sciences, Tabriz, Iran. *; h *Department of Neonatology, School of Medicine, Tehran University of Medical Sciences, Tehran, Iran.*

**Keywords:** Acetaminophen, Biomarker, Taurine, Paracetamol, Hepatotoxicity

## Abstract

Changes in plasma concentration of taurine during hospitalization of acetaminophen poisoned patients have not been studied. Hepatotoxicity is a common consequence of acetaminophen overdose that may lead to acute liver failure. Numerous biomarkers for drug-induced liver injury have been explored. All biomarkers are usually obtainable 48 h following acetaminophen overdose. We have already introduced taurine as a non-specific early biomarker of acetaminophen overdose. This study aimed to follow up changes in plasma concentration of taurine during the first three days of acetaminophen overdose. Sixty-four male patients suffering from acetaminophen overdose were selected for the study. Four blood samples were taken from the patients every 12 h. Sixty blood samples were also taken from sixty healthy humans. The plasma concentration of taurine in both groups was analyzed an already developed HPLC method. Analysis of regression showed a significant correlation between means of plasma concentrations of taurine and acetaminophen, aspartate aminotransferase, Alanine aminotransferase, glutathione peroxidase, and prothrombin time during hospitalization. The high plasma concentration of taurine, 6 h or more after acetaminophen overdose, could be a useful early indicator of liver damage.

## Introduction

Acetaminophen is a widely used analgesic and antipyretic which is available over the counter. Lack of gastrointestinal side effects and rapid absorption has made it a popular analgesic in the last four decades. As hepatotoxicity is a common complication following acetaminophen overdose, it can terminate acute liver failure (ALF). One of the most common causes of ALF in both the USA and UK is Acetaminophen (1). Nowadays, without treatment, acetaminophen overdose leads to 0.4% mortality, and in at least half of people with blood level acetaminophen above the UK standard treatment line manifests with severe liver damage (2). Cerebral edema is a major cause of death in acute acetaminophen overdose (3). Acetaminophen poisoning accounts for at least 42% of USA acute liver failure cases seen at tertiary-care centers and one-third of the death. The number of ALF cases due to acetaminophen poisoning doubled within six years (4). in recent years, the proportion of admissions involving acetaminophen increased significantly in the UK (5, 6). Another study in Canada showed that the incidence of acetaminophen overdose was 46 per 100,000 populations in the last decade (7, 8). Generation of reactive oxygen species and nitric oxide, lipid peroxidation, mitochondrial dysfunction, disruption of calcium hemostasis, and induction of apoptosis are all mechanisms suggested may be involved in acetaminophen-induced hepatotoxicity (3, 9). Numerous biomarkers for drug-induced liver injury have been explored, but less than ten are adopted or qualified as valid by the US FDA (Food and Drug Administration) (10). Increase in plasma activities of aspartate aminotransferase (AST) and alanine aminotransferase (ALT) (11, 12), glutathione peroxidase (GPx) (13, 14), lactic dehydrogenase (LDH) and hydroxybutyrate dehydrogenase (HBDH) (15, 16), glutathione S-transferase (GST) (17), argininosuccinate synthetase (18), Pentraxin 3 (19), F-protein (20), bilirubin (21), blood ammonia concentrations (22), hypoglycemia (23), prolongation of prothrombin time (PT) (24) or international normalized ratio (INR) (25, 26), and early high anion gap metabolic acidosis (27) have already been introduced as biomarkers of acetaminophen-induced liver damage. Arginase I, sorbitol dehydrogenase (SDH), ornithine carbamyltransferase (OCT), glutamate dehydrogenase, paraoxonase, malate dehydrogenase, and purine nucleoside phosphorylase have also been introduced as biomarkers of liver necrosis (28). All the biomarkers as mentioned above are usually obtainable 48 h following acetaminophen overdose. Taurine (2-aminoethane sulfonic acid) is a β-amino acid being of a sulfonic acid group substituted instead of a carboxylic acid group in the standard proteinogenic amino acids’ structures. The mammalians brain, heart, liver, neutrophils, retina, and kidneys contain high concentrations of taurine, a conditionally essential amino acid which is one of the most abundant free amino acids that is not included in protein structure (29). taurine plasma and urine concentrations vary following surgical trauma (30), muscle necrosis, stress stages, e.g. osmotic changes, anoxia, cell proliferation, brain development (31), stroke, speedy exercise (32), hepatic encephalopathy (33), and heroin addiction (34). Our study determined that mean plasma taurine level (26.4 ± 1.6 mg/L) in acetaminophen-overdose patients was significantly greater comparing to healthy humans (5.6 ± 0.2 mg/L) (*P *< 0.0001) (35). In our previous study, we were not able to show frequency of changes in plasma concentration of taurine during poisoning. Therefore, the aim of this study was to follow up those changes during the three days period following acetaminophen overdose.

## Experimental

*Methods*

Sixty-four patients (all men) suffering from acetaminophen overdose (age between 15 and 85) who had taken acetaminophen tablets seven grams or more were selected for the study after fully informed consent. None of the patients had any underlying conditions that could have affected the result, so the measured biomarkers. The Ethics Committee of Tabriz University of Medical Sciences has approved the study protocol with number 5.4.2703. The study was conducted according to the Declaration of Helsinki. Four blood samples (5 mL each) at the time of admission to the hospital, 12, 24, and 48 h following hospitalization were taken. Sixty blood samples (5ml each) were taken from sixty healthy humans (age 18- 45 years). According to the UK regimen, all the acetaminophen-poisoned patients received N-acetylcysteine as a routine regimen for managing acetaminophen overdose in Europe (36, 37). In the UK regimen, an initial dose of 150 mg/kg of body weight of NAC is infused intravenously in 200 mL of 5% dextrose over 15 min, followed by 50 mg/kg in 500 mL of 5% dextrose over four h and 100 mg/kg in one litre of 5% dextrose over the next 16 h (300 mg/kg of NAC over 20 h). Plasma samples of both groups were collected over a year and kept at −20 °C until analysis. Ghandforoush-Sattari *et al.* (38) developed a new method for analyzing taurine by HPLC using o-phtalaldehyde (OPA) and 3-mercapto-propionic acid (MPA) for derivatization of taurine and α-amino-butyric acid as internal standard with a Genesis C18 4 μm 15 cm column and disodium hydrogen phosphate 0.0125 M: acetonitrile (94:6) pH 7.2 as the mobile phase. liver enzymes level were examined by an Auto-Analyser (Hitachi^®^) using kits of Pars Daru^®^ (Iran) and prothrombin time was assessed by Dade Innovin (Dade Behring®) reagent with a Sysmex CA 1500 coagulometer in Sina Hospital laboratory. Acetaminophen levels were measured by Rostami-Hodjegan’s method (39). GPx was also measured by an enzyme-linked immune sorbent assay (ELISA) kit (CAYMAN^®^ Plasma GPx Enzyme Immunoassay). Our statistic method for analyzing data were linear regression and non-parametric student t-test (Mann-Whitney), and we used SPSS (Ver. 21) and Excel (Ver. 2007) software packages.

## Results

**Figure 1:**
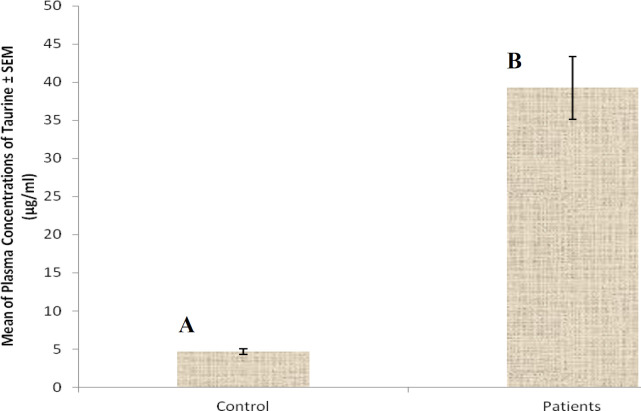
Distribution of plasma taurine levels in healthy controls (A) and acetaminophen-poisoned patients (B) (*P* < 0.0001).

**Figure 2:**
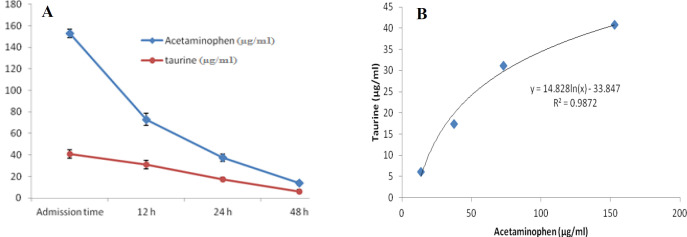
Changes of plasma concentrations of acetaminophen and taurine, during 48 h of hospitalization (A) and correlation between plasma concentrations of taurine and acetaminophen in acetaminophen-poisoned patients (B).

**Figure 3:**
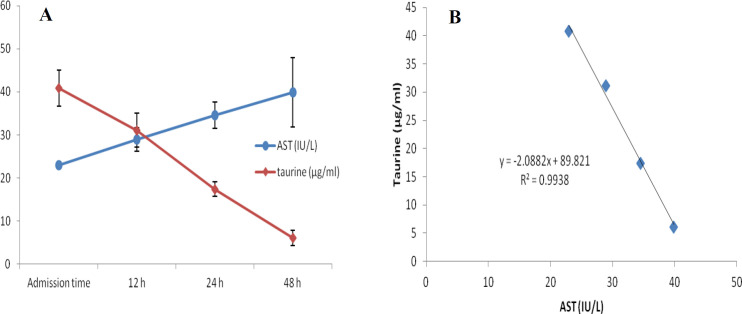
Changes of plasma concentrations of taurine and AST, during 48 h of hospitalization (A) and correlation between plasma concentrations of taurine and AST in acetaminophen-poisoned patients (B).

**Figure 4:**
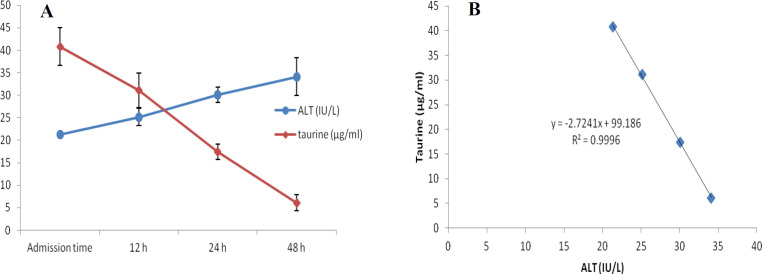
Changes of plasma concentrations of taurine and ALT, during 48 h of hospitalization (A) and correlation between plasma concentrations of taurine and ALT in acetaminophen-poisoned patients (B).

**Figure 5:**
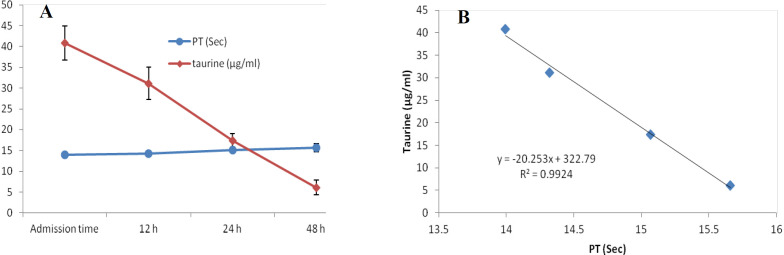
Changes of plasma concentration of taurine and PT, during 48h of hospitalization (A) and correlation between plasma concentration of taurine and PT in acetaminophen-poisoned patients (B).

**Figure 6:**
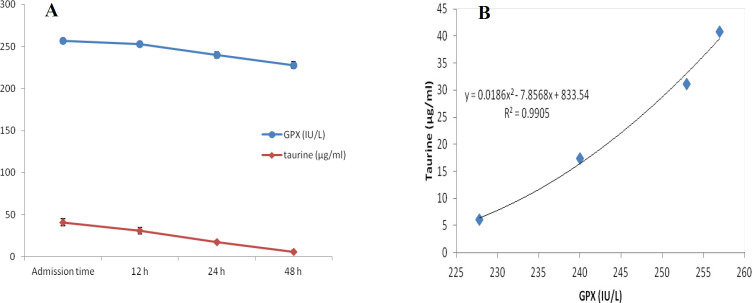
Changes of plasma concentrations of acetaminophen and GPx, during 48h of hospitalization (A) and correlation between plasma concentrations of taurine and GPx in acetaminophen-poisoned patients (B)

At first demographic characteristics of patients were examined in Table 1. We compared taurine Plasma concentrations in 64 patients with acetaminophen overdose (mean 39.2 ± 4.1 μg/mL) at the first hour of admission to the hospital with 60 healthy men (average 4.6 ± 0.2 μg/mL) by a non-parametric student t-test (Mann-Whitney) (Table 2). The mean plasma concentration of taurine in the acetaminophen overdose group was significantly more than in the control group (*P* < 0.0001). The limit of taurine plasma level concentrations in both groups was between 8.2 and 97.3 mg/L (mean 35.5) in the acetaminophen group and 1.7 and 8.9 mg/L (median 4.3) in normal healthy subjects (Figure 1). Oral acetaminophen is readily absorbed from the gastrointestinal tract, with peak plasma concentrations occurring 10-60 min after ingestion and eliminated with a plasma half-life of 2-4h (40). The severity of liver damageis directly correlated with plasma concentrations of acetaminophen. Therefore, the plasma concentrations of taurine were compared to those of acetaminophen at the same sampling times following acetaminophen overdose. At the admission time, plasma concentrations of acetaminophen and taurine were 152.9 ± 1.4 μg/mL and 40.8 ± 2.5 μg/mL, respectively. These amounts declined to 13.9 ± 1.4 μg/mL and 6.1 ± 1.7 μg/mL, respectively. As seen in Figure 2, the plasma concentration of acetaminophen correlated positively with taurine level by regression analysis during hospitalization (*R*^2^ = 0.987). Aspartate aminotransferase (AST or SGOT) is the second routine biomarker of liver damage caused by acetaminophen overdose. Plasma concentrations of AST are normally between 19 and 48 IU/L in Sina hospital of Tabriz. This study was 18-33 IU/L (mean 23.0 ± 0.75) at the admission time while it was increased to 19-97 IU/L (mean 39.9 ± 8.1) in 48 h. Analysis of regression showed a significant negative correlation between means of AST and taurine during hospitalization (R^2 ^= 0.994) (Figure 3). Alanine aminotransferase (ALT or SGPT) is the third routine biomarker in liver damage by acetaminophen overdose. Normal plasma ALT concentrations in humans are usually 7-56 IU/L in Sina hospital of Tabriz. It was 7-27 IU/L (mean 21.3 ± 0.56) at the time of admission and 19-59 IU/L (mean 34.1 ± 4.2) in 48 h in our patients. Analysis of regression showed a significant negative correlation between means of ALT and taurine during the 48 h of hospitalization (R^2 ^= 0.999) (Figure 4). The prothrombin time is a sensitive marker to assess the synthetic capacity of the liver, so it shows the severity of hepatic necrosis and used to evaluate the activity of coagulation factors (41). The normal range of Prothrombin time in a healthy human is 10.5 to 13.0 seconds. Prothrombin duration of more than 13.5s is considered abnormal. In our patients, PT was increased from 13.9 ± 0.36 (range 11-21s) at the time of admission to 15.7 ± 1.1 (range 14-22s). Analysis of regression showed a significant negative correlation between means of PT and taurine during the 48 h of hospitalization (R^2 ^= 0.992) (Figure 5). Glutathione peroxidase (GPx) is another liver enzyme that has recently been suggested to be a biomarker of liver damage (42). In our patients, it declined from 256.9 ± 2.7 (range 232.4-279.2 IU/l) to 227.8 ± 4.3 (range 211.4-247.4 IU/L). Analysis of regression revealed a remarkable positive correlation between means of GPx and taurine during the 48 h of hospitalization (R^2 ^= 0.990) (Figure 6).

## Discussion

In a previous study (35), we showed that the mean plasma concentration of taurine in the acetaminophen-overdose patients was significantly greater than of healthy humans (*P *< 0.0001). However, we could not show the changes in plasma concentration of taurine in acetaminophen poisoned patients during hospitalization because the samples were chosen randomly regardless of the sampling time. Acute liver necrosis is accompanied by elevated liver enzyme activity, AST and ALT are above1000 IU/L, followed by renal failure with an elevation of plasma creatinine levels up to 300 μmol/L (3.4 mg/100 mL) (43, 44). Kurtovic *et al.* (45) Showed that prothrombin time, serum creatinine, white cell count and abnormal potassium levels are independent mortality predictor factors due to acetaminophen-induced fulminant hepatic failure. A decrease in GPx activity has also been demonstrated by (14). Waters *et al*. (46) and Harry *et al*. (47) studies showed that taurine plasma levels rise following acetaminophen overdose. However, their study was limited to plasma concentration of taurine, and they had no concern for other biomarkers of acetaminophen poisoning. Mean peak of orally administrated acetaminophen concentration in plasma rises to approximately 70 min (48), and the half-life of paracetamol elimination from plasma is 2.5 h (49, 50). Likewise, after an acetaminophen overdose, the taurine level rises to a peak level in plasma 6 h or more and is eliminated from plasma with a half-life of 1.5 h (51). Our study showed that elevation of taurine level plasma is directly related to acetaminophen increased concentration. Liver damage due to acetaminophen overdose is followed by increased level AST and ALT concentrations (52). It was assumed that plasma AST and ALT activities simultaneously and taurine plasma elevation may directly relate. There was a significant negative correlation between plasma taurine, AST, and ALT concentrations in the acetaminophen-poisoned patients. Because the plasma concentration of taurine declines along with increased plasma levels of AST and ALT. The reason why the levels of aminotransferases and PT were so low was that all acetaminophen overdose patients were admitted to the hospital between 4 and 10 h following overdose. They were also administered NAC soon after arriving at the hospital. The most important predictor of acetaminophen-induced fulminant hepatic failure in humans is absolute prothrombin time (PT) or its international normalized equivalent (INR) (41, 53). The normal range of PT in healthy humans is 10.5 to 13.0 s, and more than this time is abnormal. In this study, plasma taurine levels and PTs have a significantly negative correlation. Following acetaminophen overdose, the plasma taurine level increases after 6–15 h and then is eliminated from plasma with a half-life of 1.5h. In acute liver failure following acetaminophen poisoning, PT increases after 48 h. Therefore, it is likely that after liver damage following acetaminophen overdose, first plasma taurine concentration rises and elevation of PT is a secondary marker. The plasma level of GPx, an indirect indicator of glutathione’s activity in the liver has already been introduced in many studies (54-56). The present study declined from 256.9 ± 2.7 to 227.8 ± 4.3 IU/L in 48 h following acetaminophen overdose. After the liver is confronted with toxins, it produces taurine, and its level increases in plasma and urine following cell damage. Hepatotoxins cause Na to pass easily through activated voltage channels, so, after Na, Cl, and water passes passively which leads to cell edema. Plasma membrane permeability increases to taurine after a hypo-osmolar condition, so taurine efflux to plasma increases (57). Increased plasma taurine after liver necrosis may lead to aminoacidemia. Acetaminophen causes GSH level decrease, which leads to diversion of cysteine synthesis away from taurine in favor of GSH, so a lower percentage of N-acetylcysteine is metabolized to taurine (58, 59). 

**Table 1:** Demographic characteristics of acetaminophen poisoned patients

**Acetaminophen-poisoned patients**		
**Age group (year)**	**Population**	**Percent**
<20	10	15.6%
21-30	30	46.9%
31-40	10	15.6%
41-50	8	12.5%
51-60	0	0%
>61	6	9.4%

**Table 2:** Biochemical blood characteristics of acetaminophen poisoned patients

**Biochemical Markers**	**Arrival sample**	**12 (h)**	**24 (h)**	**48 (h)**
Taurine (μg/mL)	39.23 ± 4.12	27.82 ± 4.02	17.96 ± 1.76	6.90 ± 1.89
Acetaminophen (μg/mL)	157.97 ± 4.09	72.95 ± 5.52	37.46 ± 3.32	13.80 ± 2.05
Creatinine (IU/L)	1.03 ± 0.06	1.02 ± 0.06	0.96 ± 0.07	0.98 ± 0.9
Urea (IU/L)	28.68 ± 1.93	26.44 ± 2.26	27.54 ± 3.84	24.56 ± 3.49
PT (s)	13.7 ± 0.4	13.9 ± 0.5	14.1 ± 0.4	14.3 ± 0.3
AST (IU/L)	36.91 ± 14.38	48.61 ± 22.80	30.29 ± 8.97	39.89 ± 14.51
ALT (IU/L)	38.65 ± 1 5.69	43.22 ± 21.12	42.57 ± 23.02	26.22 ± 5.52

## Conclusion

The high plasma concentration of taurine, 6 h or more after acetaminophen overdose, could be a useful early indicator of liver damage. While other biomarkers of acetaminophen poisoning (except serum acetaminophen concentration) increase 24 h following overdose. 

## Author contributions

MRS conceived and supervised the whole project; ASM, ZM and SH designed and performed the experiments, analyzed the data and drafted the manuscript. AO, HN and helped in performing the experimental part of the study. MRS and ASM edited the manuscript. KM helped in developing the theoretical framework.
